# Moving Toward Biomimetic Tissue-Engineered Scaffolds

**DOI:** 10.3390/nano14242028

**Published:** 2024-12-17

**Authors:** Silvia Baiguera, Lucy Di Silvio, Costantino Del Gaudio

**Affiliations:** 1 Independent Researcher; silvia.baiguera@gmail.com; 2 Centre for Oral, Clinical & Translational Sciences, Faculty of Dentistry, Oral & Craniofacial Sciences, King’s College London, London SE1 9RT, UK; lucy.di_silvio@kcl.ac.uk; 3 Italian Space Agency, Via del Politecnico snc, 00133 Rome, Italy

Advancing experimental methodologies to accurately replicate the physiological and pathological characteristics of biological tissues is pivotal in tissue engineering. Such advancements enable the development of precise in vitro and in vivo models for therapeutic research. This Special Issue, titled “Moving Toward Biomimetic Tissue-Engineered Scaffolds”, emphasizes the importance of biomimicry in designing customized scaffolds for tissue engineering applications.

A key focus in tissue engineering is replicating the microenvironment of target tissues to guide and enhance cellular responses. Ideally, scaffolds should mimic the tissue-specific extracellular matrix (ECM), be able to provide structural and biochemical cues, and support effective tissue regeneration [1]. Designing such scaffolds requires a comprehensive analysis of materials, fabrication techniques, and biological signals. Despite the challenges in replicating the hierarchical organization and intricate biochemical interactions of native ECM, a multidisciplinary approach can identify the critical features that are essential for regeneration. Scaffolds that incorporate biologically relevant properties and exhibit appropriate mechanical characteristics can potentially direct cellular behaviour to provide a conducive regenerative environment [2]. Decellularized ECM-based scaffolds are particularly promising, as combining natural biomaterials with advanced manufacturing and surface modification technologies can significantly enhance regenerative potential. This strategy broadens their therapeutic applications, offering substrates that support cellular viability and function while promoting tissue-specific regeneration [3].

Technologies play a pivotal role in the fabrication stage, leading to the development of functional microarchitectures. Key features such as porosity, pore size, interconnectivity, biosorption, and mechanical properties represent fundamental requirements for initiating a biomimetic strategy. This initial step must be further refined, for instance, by incorporating bioactive compounds through specific chemical treatments to achieve a stable binding to the scaffold or a controlled release with predefined kinetics, ensuring a precise dosage delivery. Moreover, the external environment must be considered within a comprehensive experimental design. The signals arising from culture conditions can significantly contribute to replicating physiological niches and provide additional, necessary inputs to achieve more realistic outcomes.

This rationale underpins the present Special Issue, which aims to deliver a focused, albeit partial, overview of the research conducted thus far in tissue engineering and regenerative medicine. Additionally, it seeks to provide perspectives on potential future projects in the field.

A biomimetic strategy remains one of the principal drivers for designing innovative investigative protocols. To this end, an effective approach must appropriately integrate the properties of biomaterials, cellular responses, and signalling pathways. This Special Issue offers a balanced mix of original research and review articles, alongside a perspective study addressing critical aspects of this topic.

Notably, three research articles focus on electrospun scaffolds as effective tools to replicate tissue-specific characteristics or promote tailored cellular responses. Rivera-Torres et al. [4] developed composite scaffolds composed of poly(vinyl alcohol) and bioglass type 58S to investigate the spontaneous activity of chick embryonic cardiomyocytes, examining how the inorganic component content affects synchronous beating patterns. Sousa de Almeida et al. [5] explored the role of polyurethane electrospun nanofibers in nanoparticle uptake by human fibroblasts and epithelial cells, revealing that substrate properties significantly influence cellular behaviour. These findings provide critical insights for designing disease models and nanoparticle-based therapies. Niu and Galluzzi [6] studied layered tubular hyaluronic acid-collagen scaffolds that mimic the intima and media layers of blood vessels. Their results demonstrate that the complete endothelialisation of mouse vascular endothelial cells along the inner wall facilitates vascular smooth muscle cell infiltration and alignment, conforming to the hierarchical architecture of the nanofibrous scaffold. This structure supports the maintenance of contractile phenotypes and natural growth behaviour. Sassi et al. [7] expanded the scope of tissue engineering by investigating decellularized rat livers. Their study employed a custom-designed bioreactor to sustain dynamic culture conditions, enabling non-invasive analyses of cell viability, distribution, and function.

The review articles critically evaluate the existing data, emphasizing the importance of biomimetic procedures in achieving functional tissue regeneration. Górnicki et al. [8] present a survey on various scaffolds that facilitate three-dimensional cell cultures, a well-established prerequisite for accurately mimicking biological tissues. Roato et al. [9] analyze the role of scaffolds in periodontal tissue engineering, with an emphasis on mechanical stimulation to enhance the reliability of the results. Cun and Hosta-Rigau [10] examine cellular responses to scaffold topography, focusing on the behaviour of mesenchymal stem cells. They emphasize how surface features, including roughness, patterns, and porosity, regulate cell fate. Although a definitive correlation between topographical features and cellular responses remains unestablished, this approach offers a complementary or alternative strategy to the biochemical modulation of stem cell differentiation. An essential aspect of implantable devices is their immunological impact. Corsi et al. [11] address this by investigating macrophage polarization through bioactive metallic and ceramic nanoparticles, which hold promise as tools for promoting skeletal muscle regeneration. Politi et al. [12] extend this discussion by reviewing biofunctionalized electrospun mats and their potential for developing advanced smart scaffolds.

Furthermore, Baiguera et al. [13] present a forward-looking perspective on 3D-printing biomimetic scaffolds for skeletal muscle regeneration. They propose an experimental approach, utilizing additive manufacturing techniques, specifically stereolithography, to process hydrogels enriched with a decellularized extracellular matrix.

In summary, the contributions presented in this Special Issue highlight the immense potential of biomimetics for designing and fabricating scaffolds that advance tissue engineering. Enhancing the performance of biomedical devices to drive functional tissue regeneration is a pressing need to achieve the goal of improving the outcomes of novel therapeutic protocols.
